# Significance of CA125 Monitoring during Maintenance Treatment with Poly(ADP-Ribose) Polymerase Inhibitor in Ovarian Cancer after First-Line Chemotherapy: Multicenter, Observational Study

**DOI:** 10.32604/or.2025.068609

**Published:** 2025-10-22

**Authors:** Szymon Piątek, Anna Dańska-Bidzińska, Paweł Derlatka, Bartosz Szymanowski, Renata Duchnowska, Aleksandra Zielińska, Natalia Sawicka, Aleksander Gorzeń, Wojciech Michalski, Mariusz Bidziński

**Affiliations:** 1 Department of Gynecologic Oncology, Maria Sklodowska-Curie National Research Institute of Oncology, Warsaw, 02-781, Poland; 2 Department of Obstetrics and Gynecology, Medical University of Warsaw, Warsaw, 02-091, Poland; 3 Department of Oncology, Military Institute of Medicine—National Research Institute, Warsaw, 04-141, Poland; 4 Department of Obstetrics, Gynecology and Gynecological Oncology, Medical University of Warsaw, Warsaw, 02-091, Poland; 5 Faculty of Medicine, Medical University of Warsaw, Warsaw, 02-091, Poland; 6 Department of Biomedical Statistics, Maria Sklodowska-Curie National Research Institute of Oncology, Warsaw, 02-781, Poland

**Keywords:** Ovarian cancer, cancer antigen 125 surveillance, recurrence, poly(ADP-ribose) polymerase inhibitor

## Abstract

**Objectives:**

Monitoring of Cancer Antigen 125 (CA125) during ovarian cancer (OC) maintenance treatment with poly(ADP-ribose) polymerase inhibitors (PARPis) may be insufficient when using Gynecologic Cancer Intergroup (GCIG) biochemical progression criteria. This study aimed to evaluate the usefulness of CA125 monitoring in detecting OC recurrence during PARPis maintenance treatment.

**Methods:**

This multicenter retrospective cohort study included patients with primary OC who achieved complete or partial response after first-line platinum-based chemotherapy followed by PARPis maintenance treatment. Progression was defined using Response Evaluation Criteria in Solid Tumors (RECIST) and GCIG biochemical criteria. New biochemical progression definitions, based on CA125 nadir determined using receiver operating characteristic (ROC) curve analysis, were proposed. Concordance between radiological and biochemical progression was assessed.

**Results:**

Of 142 patients, progression was detected in 54 (38.03%) and 29 (20.42%) using RECIST and GCIG criteria, respectively. The sensitivity, specificity, positive predictive value (PPV), and negative predictive value (NPV) of the GCIG criteria were 53.70% [95% confidence interval (CI): 39.61%–67.38%], 100.00% [95% CI:95.91%–100.00%], 100.00% [95%CI: 88.10%–100.00%] and 77.88% [95% CI: 72.54%–82.43%], respectively. A cut-off of 1.59× nadir achieved 88.90% sensitivity and 87.20% specificity [Area Under Curve (AUC): 91.10%, 95% CI: 84.70%–97.40%] with a false positive rate (FPR) of 12.67%. Defining biochemical progression as an increase in CA125 of ≥3× nadir achieved sensitivity, specificity, PPV, NPV, and FPR of 79.63% [95% CI: 66.47%–89.37%], 98.86% [95% CI: 93.83%–99.97%], 97.73% [95% CI: 85.91%–99.67%], 88.78% [95% CI: 82.35%–93.06%], and 1.14%, respectively. Diagnostic accuracy was higher using the ≥3× nadir criterion compared with GCIG definition (91.55% vs. 82.39%).

**Conclusion:**

GCIG biochemical progression criteria during PARPis maintenance treatment after first-line chemotherapy missed 46.3% of progressing patients. A new criterion—CA125 ≥3× nadir—improves sensitivity and NPV, while maintaining high specificity, offering a simple and practical approach for clinical implementation.

## 1 Introduction

The effectiveness of poly(ADP-ribose) polymerase inhibitors (PARPis) in the treatment of platinum-sensitive ovarian cancer (OC) has been confirmed in several randomized phase III trials. Pujade-Lauraine et al. demonstrated that olaparib maintenance therapy significantly improved progression-free survival (PFS) in patients with platinum-sensitive, relapsed OC harboring a BRCA1/2 mutation [1]. Coleman et al., investigating rucaparib in relapsed OC, confirmed that the greatest clinical benefit was observed in patients with BRCA1/2 mutations (median PFS: 16.6 vs. 5.6 months). Moreover, patients with homologous recombination deficiency (HRD) also derived benefit from therapy (median PFS: 13.6 vs. 5.4 months) [2]. Mirza et al. reported that niraparib conferred significant clinical benefit irrespective of BRCA germline mutation status and even independently of homologous recombination status in patients with relapsed ovarian cancer [3]. Patients with HRD-negative tumors experienced improved outcomes with niraparib treatment (median PFS: 6.9 vs. 3.8 months) [3]. The efficacy of PARPis has also been confirmed in patients with newly diagnosed advanced OC. Veliparib maintenance therapy following first-line chemotherapy resulted in significantly prolonged progression-free survival [4].

Maintenance treatment with PARPis has emerged as a breakthrough, significantly prolonging survival without compromising quality of life [5–7]. While controversy persists regarding the role of hyperthermic intraperitoneal chemotherapy and adjuvant intraperitoneal chemotherapy, the use of PARPis following completion of chemotherapy is well established [8,9]. European Society of Medical Oncology recommendations and National Comprehensive Cancer Network guidelines are consistent in this regard—patients with platinum-sensitive OC should receive PARPis in primary or recurrent disease [8,9].

However, PARPi therapy is not without adverse effects. Due to treatment-related toxicity, dose reductions are required in 30%–70% of patients [10]. The most common side effects include fatigue, nausea, and anemia [10]. Of particular concern is the potential for PARPis to induce resistance to subsequent chemotherapy in patients who were initially platinum-sensitive [11,12]. The mechanisms of resistance are multifactorial and not yet fully understood [13]. Nonetheless, prolonged exposure to PARPis after loss of therapeutic efficacy may increase the risk of developing such resistance. Therefore, early detection of relapse is needed to discontinue therapy at the point when the patient no longer derives clinical benefit.

Before the introduction of PARPis, monitoring of cancer antigen 125 (CA125) serum levels during follow-up was a standard method for assessing disease status. Rising CA125 levels—even within the normal range—were shown to precede recurrence of OC [14]. However, recently published study evaluating PARPis in the treatment of recurrent OC has raised questions about the reliability of CA125 in detecting subsequent recurrence [15]. It was shown that almost 50% of patients with disease progression showed no corresponding increase in CA125 concentration [15,16].

The aim of this study was to evaluate the utility of CA125 monitoring in detecting ovarian cancer recurrence during maintenance treatment with PARPis following first-line chemotherapy.

## 2 Materials and Methods

### 2.1 Study Design

This multicenter, observational study was conducted at the following institutions: the Department of Gynecologic Oncology, Maria Sklodowska-Curie National Research Institute of Oncology (MSCNRIO); the 2nd Department of Obstetrics and Gynecology, Medical University of Warsaw (DOG-MUW); the Department of Oncology, Military Institute of Medicine—National Research Institute (DOMIM); and the Department of Obstetrics, Gynecology and Gynecological Oncology, Medical University of Warsaw (DOGGO-MUW). All consecutive patients with primary OC treated between 01 January 2021 and 31 December 2022, who received PARPi maintenance treatment following first-line chemotherapy were eligible for the study. During this period, two PARPis, olaparib and niraparib, were reimbursed in Poland. Eligibility criteria for enrollment in the PARPi program included: (1) histologically confirmed high-grade epithelial OC, (2) complete or partial response to platinum-based chemotherapy, (3) International Federation of Gynecology and Obstetrics (FIGO) stage III or IV [17] at initial diagnosis, regardless of homologous recombination (HR) status.

Patients who had previously received PARPi therapy, as well as those with normal or unknown pre-treatment CA125 were excluded from the study.

Both PARPis were permitted in first-line treatment for patients meeting criteria 1–3; however, olaparib was restricted to patients with BRCA1/2 mutations and HR deficiency, while niraparib could be used regardless of BRCA 1/2 and HR status. The choice between olaparib or niraparib was at the discretion of the treating physician. Maintenance treatment was initiated within 12 weeks of the final cycle of chemotherapy. For patients with a partial response, treatment with PARPis continued until disease progression. In patients with a complete response, treatment was administered until disease progression, unacceptable toxicity, or for a maximum duration of 2 years (olaparib) or 3 years (niraparib).

### 2.2 Surveillance

Treatment monitoring included serial CA125 serum measurements and computed tomography (CT) imaging. CA125 levels were assessed every month. Imaging of the chest, abdomen and pelvis was performed at least every 6 months, or earlier if clinical or biochemical progression was suspected.

Progressive disease (PD) was defined according to Response Evaluation Criteria in Solid Tumor (RECIST) version 1.1 criteria [18]. Biochemical (marker) progression was diagnosed based on Gynecologic Cancer Intergroup (GCIG) criteria [19].

### 2.3 Development of New CA125 Progression Criteria

To optimize and personalize the definition of CA125 progression, new criteria were proposed independently of achieving CA125 normal range after chemotherapy. Previous studies have indicated that the CA125 nadir is a significant prognostic factor for ovarian cancer progression [20,21]. Therefore, the CA125 nadir was selected as the reference point for receiver operating characteristic (ROC) curve analysis, which was used to determine the optimal cut-off thresholds for defining progression.

### 2.4 Statistical Analysis

Descriptive statistics were performed for both continuous and categorical variables. Sensitivity, specificity, positive predictive value (PPV), negative predictive value (NPV), accuracy, positive likelihood ratio, and negative likelihood ratio of biochemical progression were assessed relative to RECIST-based progression. Kaplan–Meier survival curves and the log-rank test were used to compare patients with vs. without CA125 progression. Statistical analyses were conducted using SPSS Statistics version 26.0 (IBM Corp., Armonk, NY, USA).

### 2.5 Ethical Approval

This study was approved by Bioethical Committee of the Medical University of Warsaw (No. AKBE/117/2024). All procedures were conducted according to the Declaration of Helsinki for Medical Research involving Human Subjects. The clinical decisions concerning the treatment were not influenced by the purpose of this paper. Informed consent to participate in this study has been waived by Bioethical Committee of the Medical University of Warsaw. Besides, this study was prepared according to the STROBE guideline, and a STROBE checklist was provided. Please see Supplementary Material S1 for more details.

## 3 Results

**Figure 1:**
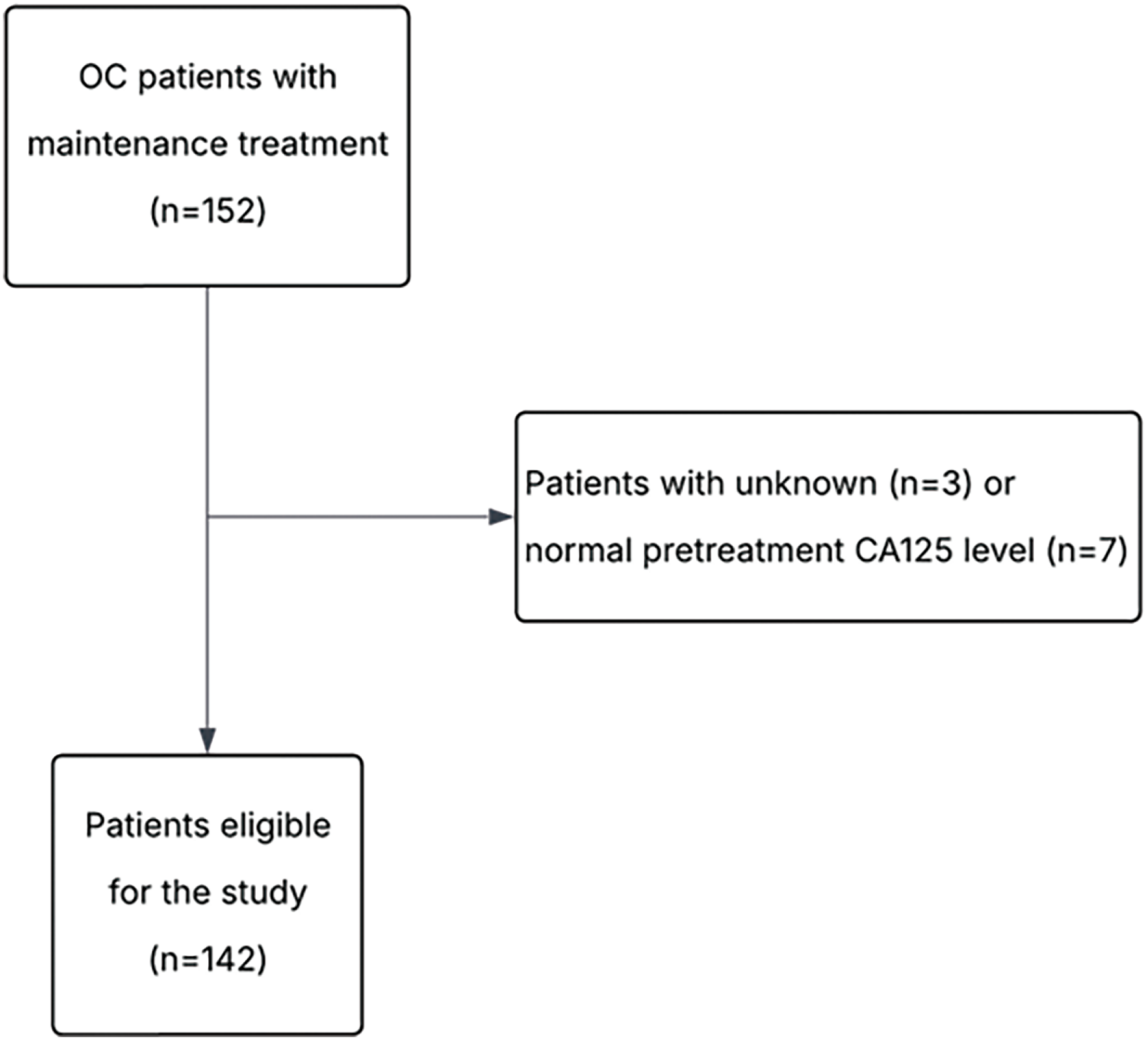
Flowchart of the study. OC, ovarian cancer; CA125, cancer antigen 125

A total of 142 consecutive patients were included in the study (Fig. 1). The distribution of patients across participating centers was as follows: 66 (46.5%) from MSCNRIO, 40 (28.2%) from DOG-MUW, 31 (21.8%) from DOMIM and 5 (3.5%) patients from DOGGO-MUW. Patient characteristics are summarized in Table 1. Mean follow-up was 23.4 months (range: 1.4–53.6 months).

**Table 1:** Patient characteristics

Variable	Study group (n = 142)
Age, mean (range), years	58.3 (35.3–79.6)
BMI, mean (range)	25.7 (16.4–43.7)
BRCA 1/2 mutation	68 (47.9%)
Non-BRCA, HRD	14 (9.9%)
Histology	
Serous high grade	136 (95.8%)
Endometrial high-grade	4 (2.8%)
Carcinosarcoma	1 (0.7%)
Clear cell	1 (0.7%)
FIGO stage	
IIIA	12 (8.4%)
IIIB	24 (16.9%)
IIIC	68 (47.9%)
IV (unspecified)	4 (2.8%)
IVA	10 (7.0%)
IVB	24 (16.9%)
Response to chemotherapy	
Complete	78 (54.9%)
Partial	64 (45.1%)
Pretreatment CA125, mean (range) U/mL	1543.8 (37.2–15,217.0)
Nadir CA-125, U/mL	
≤35	127 (89.4%)
>35	15 (10.6%)
Maintenance treatment	
Niraparib	64 (45.1%)
Olaparib	42 (29.5%)
Olaparib + bevacizumab	36 (25.4%)

Note: BMI, body mass index; BRCA, breast cancer gene; HRD, homologous recombination deficiency; FIGO, international federation of gynecology and obstetrics; CA125, cancer antigen 125.

PD according to RECIST and biochemical GCIG criteria was diagnosed in 54 (38.03%) and 29 (20.42%) patients, respectively (Table 2). The sensitivity, specificity, PPV, NPV of the GCIG marker progression criteria were 53.70% [95% confidence interval (CI): 39.61%–67.38%], 100.00% [95% CI: 95.91%–100.00%], 100.00% [95% CI: 88.10%–100.00%], 77.88% [95%CI: 72.54%–82.43%], respectively. Median progression-free survival did not differ significantly between patients with CA125 progression vs. without CA125 progression (20.1 months vs. 23.3 months; log rank test, *p* = 0.18). No statistically significant differences were observed between patients with vs. without CA125 progression in terms of FIGO stage (*p* = 0.69), BRCA1/2 mutation status (*p* = 0.17), type of cytoreductive surgery (*p* = 0.95), residual disease after surgery (*p* = 0.35), response to chemotherapy (*p* = 0.74) and type of PARPi administered (*p* = 0.29).

**Table 2:** Relationship between RECIST PD and GCIG biochemical criteria for progression

CA125 progression according to GCIG	RECIST PD		Total
		No	Yes
No	88 (100.00%)	25 (46.30%)	113 (79.58%)
Yes	0	29 (53.70%)	29 (20.42%)
Total	88 (100.00%)	54 (100.00%)	142 (100.00%)

Note: GCIG, gynecologic cancer intergroup; PD, progressive disease; RECIST, response evaluation criteria in solid tumor; CA125, cancer antigen 125.

**Figure 2:**
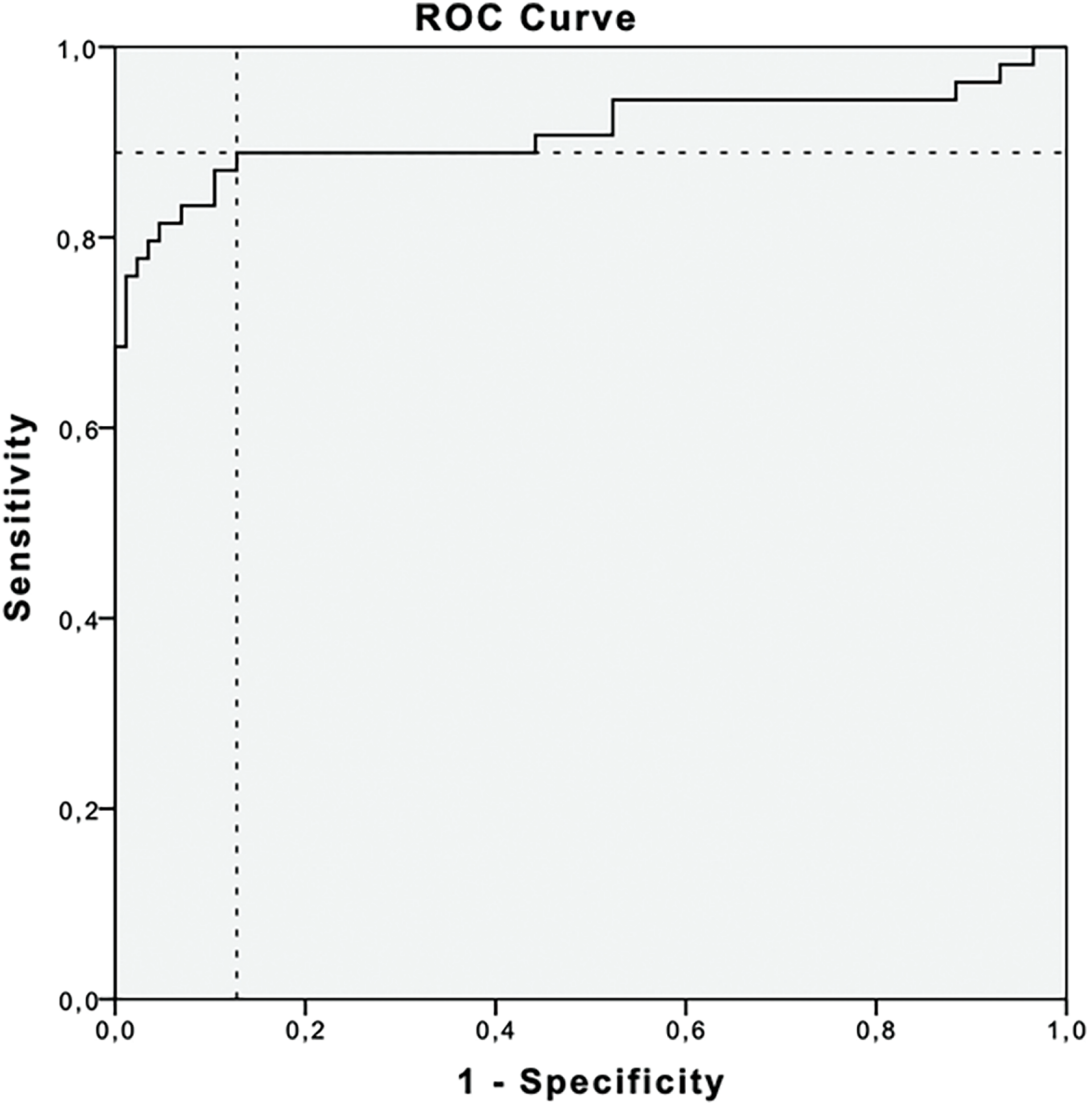
Receiver operating curve (ROC) and area under the curve (AUC) for evaluating prognostic value of the CA-125 nadir. Cut-off level for 1.59× nadir was found to have 88.9% sensitivity and 87.2% specificity (AUC: 91.1%, 95% CI: 84.7%–97.4%)

Given the prognostic significance of the CA125 nadir, new criteria for marker progression were evaluated using ROC curve analysis (Fig. 2). A cut-off value of 1.59× nadir demonstrated a sensitivity of 88.9% and specificity of 87.2% (AUC: 91.1%; 95% CI: 84.7%–97.4%). However, given the limited practicality of using this exact threshold in clinical settings, simplified criteria using 2× and 3× the CA125 nadir level were assessed.

Progression based on a CA125 increase ≥2× nadir was observed in 45 patients with PD, compared to 29 patients meeting standard GCIG criteria (Table 3). The sensitivity, specificity, PPV, and NPV of the ≥2× nadir level were 83.33% (95% CI: 70.71%–92.08%), 93.18% (95% CI: 85.75%–97.46%), 88.24% (95% CI: 77.44%–94.25%), and 90.11% (95% CI: 83.35%–94.31%), respectively.

**Table 3:** Relationship between RECIST PD and new proposed ≥2× nadir CA125 progression

≥2× nadir CA125 progression	RECIST PD		Total
		No	Yes
No	82 (93.18%)	9 (16.66%)	91 (64.08%)
Yes	6 (6.82%)	45 (83.34%)	51 (35.92%)
Total	88 (100.00%)	54 (100.00%)	142 (100.00%)

Note: RECIST, response evaluation criteria in solid tumor; PD, progression disease; CA125, cancer antigen 125.

An increase in CA125 levels to ≥3× the nadir value was observed in 43 patients with PD (Table 4). The corresponding sensitivity, specificity, PPV, and NPV were 79.63% (95% CI: 66.47%–89.37%), 98.86% (95% CI: 93.83%–99.97%), 97.73% (95% CI: 85.91%–99.67%), and 88.78% (95% CI: 82.35%–93.06%), respectively.

**Table 4:** Relationship between RECIST PD and new proposed ≥3× nadir CA125 progression

≥3× nadir CA125 progression	RECIST PD		Total
		No	Yes
No	87 (98.86%)	11 (20.37%)	98 (69.01%)
Yes	1 (1.14%)	43 (79.63%)	44 (30.99%)
Total	88 (100.00%)	54 (100.00%)	142 (100.00%)

Note: RECIST, response evaluation criteria in solid tumor; PD, progression disease; CA125, cancer antigen 125.

Among all evaluated criteria, the ≥3× nadir threshold demonstrated the highest overall diagnostic accuracy for predicting progression compared to both the GCIG and 2× nadir criteria (Table 5). Notably, the false positive rate for the 1.59× nadir threshold was 12.67%, for the ≥2× nadir threshold, it was 6.82%, whereas for the ≥3× nadir threshold, a false positive result was observed in only one (1.14%) patient.

**Table 5:** Comparison of different criteria of OC biochemical progression based on CA125 nadir level

Diagnostic parameter	GCIG progression	≥1.59× nadir CA125 progression	≥2× nadir CA125 progression	≥3× nadir CA125 progression
Accuracy	82.39%	88.03%	89.44%	91.55%
Positive likelihood ratio	NA	7.11	12.22	70.07
Negative likelihood ratio	0.46	0.13	0.18	0.21
False positive rate	0%	12.67%	6.82%	1.14%

Note: NA, not applicable due to lack of any false positive results; CA125, cancer antigen 125; OC, ovarian cancer; GCIG, gynecologic cancer intergroup.

## 4 Discussion

GCIG marker criteria for OC progression were concordant with radiologic progression only in 53.7% of patients treated with PARPis. Similarly, poor concordance between RECIST PD and GCIG CA125 progression has been reported in patients with platinum-resistant OC [22] and in those with platinum-sensitive recurrent disease [23]. No significant clinical, pathological, or genetic differences were observed between patients with and without biochemical progression. Despite the high rate of discordance between RECIST and GCIG biochemical criteria, 52 out of 54 patients (96.3%) with PD exhibited an increase in CA125 serum concentration at the moment of progression compared to nadir levels. Therefore, we attempted to create more individualized definition of CA125 progression.

The majority of patients (n = 127; 89.4%) achieved a CA125 nadir below the upper limit of the normal range (from 3.6 to 34.55 U/mL) following chemotherapy and prior to initiation of maintenance treatment with PARPis. According to current GCIG criteria, these patients can be diagnosed with biochemical progression once CA125 levels exceed 70 U/mL. However, in patients with low post-treatment nadir values, reaching this threshold often reflects a multiple-fold increase in CA125 concentration; while for patients with nadir values slightly below the upper limit of normal range, a relatively modest increase is enough to meet the GCIG criteria for CA125 progression.

To address this inconsistency, new criteria were proposed, independent of obtaining the CA125 normal range after chemotherapy. It has been demonstrated that a rise in CA125 from the nadir possesses prognostic significance for disease progression. An increase of 5 or 10 U/mL above the nadir has been associated with an increased risk of recurrence and decreased survival in patients achieving a complete response to therapy [14]. Du et al. reported that a CA125 level equal to twice the nadir may enable earlier detection of relapsed ovarian cancer [24]. Wang et al., using receiver operating characteristic (ROC) analysis, identified a CA125 level of 1.68× nadir as an indicator of recurrent disease [25]. In our cohort, the corresponding cut-off value was 1.59. Although an increase in CA125 to ≥1.59 times the nadir demonstrated substantially higher sensitivity than the GCIG definition (88.9% vs. 53.7%), it was associated with an unacceptably high false positive rate of 12.67%. Given that the decision to discontinue maintenance therapy due to disease progression requires high degree of diagnostic certainty, minimizing the risk of false-positive rates is essential.

At the same time, clinicians require a biomarker threshold that is both reliable and straightforward to apply in routine clinical application. The 1.59× nadir threshold, while sensitive, is unlikely to be feasible for implementation. Moreover, its false-positive rate of 12.67% significantly limits its clinical utility. Therefore, we evaluated two alternative thresholds: a 2-fold and a 3-fold increase from the nadir, which are expected to offer improved reliability and feasibility for implementation in everyday clinical settings.

Rising CA125 serum level is associated with OC progression [14]. However, Rustin et al. showed that early initiating chemotherapy based on CA125 serum elevation did not prolong overall survival and was associated with treatment-related toxicity [26]. Therefore, routine CA125 monitoring was not considered mandatory during surveillance. However, at that time, patients were not receiving maintenance treatment.

In the current era, the therapeutic landscape of OC has evolved significantly, with PARPis treatment becoming the standard of care for patients with platinum-sensitive disease [8,9]. In this context, monitoring CA125 assumes a new and critical role not only as an indicator of disease recurrence, but also as a surrogate marker of loss of effectiveness of maintenance therapy.

This issue becomes particularly important when rising CA125 levels are observed over time, while radiologic criteria for PD are not met for several months (Table 6). In such cases, continuation of PARPi therapy becomes questionable, as patients are potentially exposed to toxicity without deriving further benefit. The dilemma is compounded in patients with partial response to initial treatment, where residual after disease remains post-chemotherapy. According to RECIST, progression cannot be confirmed until there is at least a 20% increase in the size of target lesions, which may take time to manifest.

**Table 6:** Patients with progression (according to RECIST and CA125) with different patterns of rising CA125 serum concentration. Underlined values indicate the moment of recurrence according to RECIST criteria. Maintenance treatment may continue for several months after the marker progression criteria are met. However, an increasing trend was already seen earlier, suggesting a loss of effectiveness of maintenance PARPis treatment

Patient	CA125 serum concentration [IU/mL]									
	Nadir	(−2) months	(−1) month	0 GCIG PD	(+1) month	(+2) months	(+3) months	(+4) months	(+5) months	(+6) months
**1**	18.0	39.3	56.0	77.2	83.6	90.1	143.7	201.9	268.5	**333.8**
**2**	16.9	37.7	56.9	461.0	579.0	944.0	**1011.0**			
**3**	4.6	34.9	57.1	117.1	**309.6**					
**4**	13.4	42.2	50.6	106.1	105.3	140.7	**1039.0**			
**5**	61.5	97.9	119.8	129.6	142.1	**194.7**				

Note: CA125, cancer antigen 125; RECIST, response evaluation criteria in solid tumors; PARPis, poly(ADP-ribose) polymerase inhibitors; GCIG, gynecologic cancer intergroup; PD, progression disease.

Clinical assessment can be inconclusive. Symptoms of recurrence may be subtle or attributed to ongoing PARPi maintenance therapy. Some patients may deliberately underreport symptoms due to concerns about discontinuation of the treatment. This creates a clinical impasse: the benefit of continued PARPi use becomes doubtful, while the burden of toxicity is certain.

An additional problem related to PARPi treatment in case of increasing CA125 concentration is the financial aspect. Currently, it is one of the most expensive forms of therapy for OC [27,28]. Therefore, reliable biomarkers are needed to assess the effectiveness of treatment and optimize the costs of this therapy.

These challenges highlight the unmet need for reliable and timely biomarkers to support clinical decision-making during maintenance therapy. A personalized approach to CA125 monitoring, integrating nadir-based criteria, may provide a more accurate reflection of treatment failure and facilitate more appropriate timing for imaging studies and therapeutic interventions. Establishing the optimal CA125 cutoff for defining disease progression is crucial. A threshold that is too low, such as 1.59× nadir, carries a high false positive rate (12.67%), which may lead to premature discontinuation of an effective therapy or unnecessary diagnostic procedures, thereby increasing costs and patient anxiety. Conversely, a higher cutoff, such as ≥3× nadir, markedly reduces the risk of false positives (only one patient in our cohort), allowing more confident clinical decisions without compromising diagnostic accuracy.

Thus, refining CA125-based criteria may not only improve patient care by ensuring timely identification of true progression but also help rationalize healthcare expenditures, particularly in the era of high-cost maintenance therapies.

Some limitations of the study should be highlighted. First, its retrospective design may introduce selection bias and confounding variables, as well as information bias resulting from variability in data recording, diagnostic assessments, and CA125 measurement practices across participating centers. A total of 106 (74.65%) patients received PARPi-only maintenance therapy, while 36 (25.35%) patients received olaparib and bevacizumab. Another limitation was the lack of determination of HR status in all patients without BRCA1/2 mutations; among 60 such patients, HRD testing was performed in 34 (56.67%) patients, whereas in 26 (43.33%) patients no HRD test was done. Although multicenter in design, the study included 142 newly diagnosed OC patients, all of whom were caucasian and platinum-sensitive. These variables may limit the generalizability of the study’s findings. A larger cohort may be needed to validate its broader applicability in predicting loss of efficacy of PARPi and ovarian cancer recurrence. As PARPi are also approved in relapsed OC, it would be reasonable to evaluate CA125 monitoring in patients after subsequent lines of systemic treatment receiving maintenance therapy. A larger number of patients could also allow for the identification of subgroups of patients based on the location and extent of recurrence. Analysis of serum concentration of CA-125 may have particular value in patients with slow-growing tumors or oligometastatic disease during PARPi maintenance, when its use beyond progression may be beneficial.

## 5 Conclusions

The GCIG criteria for CA125 progression were established in 2005, prior to the era of maintenance treatment with PARPis. This study demonstrated that almost 42% of patients with PD did not meet the GCIG definition of biochemical progression. Nevertheless, rising CA125 serum concentrations were observed, indicating the need to develop new marker progression criteria adapted to the PARPi era in order to preserve the clinical utility of CA125 monitoring. Otherwise, the value of CA125 assessment in detecting ovarian cancer recurrence will be substantially limited.

Among the thresholds evaluated, an increase in CA125 concentration to at least three times the nadir showed the best concordance with RECIST-defined progression. This definition provides high specificity and positive predictive value, minimizes the risk of false positives, and is simple to implement in routine clinical practice. Importantly, such nadir-based criteria may facilitate more accurate identification of loss of treatment efficacy, thereby preventing unnecessary continuation of expensive maintenance therapy and avoiding prolonged exposure to treatment-related toxicity.

These findings support the clinical usefulness of CA125 monitoring when interpreted in relation to the nadir level, particularly in the context of maintenance therapy with PARPis. However, the results should be interpreted with caution given the retrospective design, the limited sample size, and incomplete homologous recombination status assessment in a subset of patients.

Further multicenter prospective studies are warranted to validate the proposed nadir-based criteria across broader patient populations and treatment settings, including patients receiving PARPi after relapse. Future research should also explore whether specific patient subgroups, such as those with oligometastatic or slow-growing disease, derive particular benefit from refined CA125 monitoring strategies.

In conclusion, adapting CA125 progression criteria to the PARPi era may improve the accuracy of disease monitoring, optimize therapeutic decision-making, and contribute to more rational allocation of healthcare resources.

## Supplementary Materials



## Data Availability

The data that support the findings of this study are available from the Corresponding Author, Anna Dańska-Bidzińska, upon reasonable request.

## Supplementary Materials

The supplementary material is available online at https://www.techscience.com/doi/10.32604/or.2025.068609/s1.
